# Clinical translation of patient-derived tumour organoids- bottlenecks and strategies

**DOI:** 10.1186/s40364-022-00356-6

**Published:** 2022-03-10

**Authors:** Malia Alexandra Foo, Mingliang You, Shing Leng Chan, Gautam Sethi, Glenn K. Bonney, Wei-Peng Yong, Edward Kai-Hua Chow, Eliza Li Shan Fong, Lingzhi Wang, Boon-Cher Goh

**Affiliations:** 1 Cancer Science Institute of Singapore, National University of Singapore, Singapore, Singapore; 2 Hangzhou Cancer Institute, Key Laboratory of Clinical Cancer Pharmacology and Toxicology Research of Zhejiang Province, Hangzhou, 31002 China; 3 Affiliated Hangzhou Cancer Hospital, Zhejiang University School of Medicine, Hangzhou, 31002 China; 4 Department of Surgery, National University Hospital, Singapore, Singapore; 5 NUS Centre for Cancer Research (N2CR), Yong Loo Lin School of Medicine, National University of Singapore, Singapore, Singapore; 6 Department of Pharmacology, Yong Loo Lin School of Medicine, National University of Singapore, Singapore, Singapore; 7 Department of Haematology-Oncology, National University Hospital, National University Health System, Singapore, Singapore; 8 Department of Biomedical Engineering, National University of Singapore, Singapore, Singapore

**Keywords:** Tumour, Organoid, Precision, Medicine, Three-Dimensional (3D)

## Abstract

Multiple three-dimensional (3D) tumour organoid models assisted by multi-omics and Artificial Intelligence (AI) have contributed greatly to preclinical drug development and precision medicine. The intrinsic ability to maintain genetic and phenotypic heterogeneity of tumours allows for the reconciliation of shortcomings in traditional cancer models. While their utility in preclinical studies have been well established, little progress has been made in translational research and clinical trials. In this review, we identify the major bottlenecks preventing patient-derived tumour organoids (PDTOs) from being used in clinical setting. Unsuitable methods of tissue acquisition, disparities in establishment rates and a lengthy timeline are the limiting factors for use of PDTOs in clinical application. Potential strategies to overcome this include liquid biopsies via circulating tumour cells (CTCs), an automated organoid platform and optical metabolic imaging (OMI). These proposed solutions accelerate and optimize the workflow of a clinical organoid drug screening. As such, PDTOs have the potential for potential applications in clinical oncology to improve patient outcomes. If remarkable progress is made, cancer patients can finally benefit from this revolutionary technology.

## Introduction

Cancer is a leading cause of death globally, responsible for 1 in every 6 deaths, and an approximate 10 million deaths in 2020 alone [1]. According to the World Health Organization (WHO), the most common causes of mortality were lung, colorectal, liver, stomach and breast cancer. Despite being the most frequently diagnosed cancers, current treatment remains ineffective in achieving curative effects in certain patients, causing their demise. This can be attributed to the “one-size-fits-all” standard of care for anti-cancer treatment which does not account for heterogeneity, rendering it ineffective and obsolete. Inter-patient heterogeneity and intra-patient heterogeneity are the key reasons for therapeutic failure for standardized anti-cancer treatment [2, 3]. Standard chemotherapy drugs may not be effective for all patients for this reason.

The rise of precision medicine is an emerging approach to the targeted selection of optimal treatment options based on each individual’s genes, environment and lifestyle. Precision medicine, in the context of cancer treatment, is to identify effective therapeutic strategies specific for every patient [4], by using targeted therapies that are less invasive and morbid than standard treatment regimens yet achieving good outcomes. Organoid technology is one that holds significant potential in realizing this goal.

**Fig. 1:**
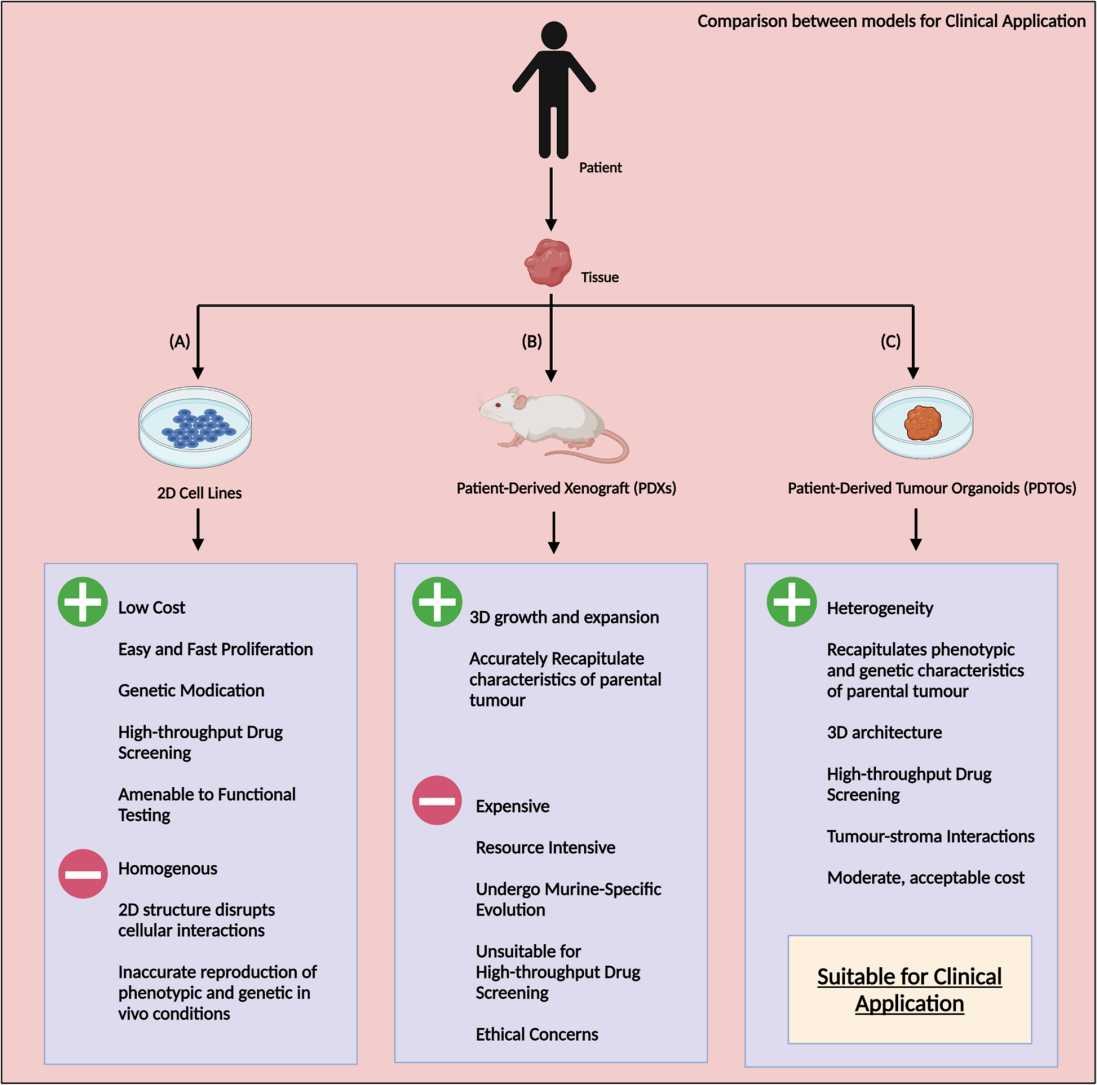
Comparison of Cell Lines, Patient-Derived Xenografts (PDXs) and Patient-Derived Tumour Organoids (PDTOs). **A**: 2D cell line model; **B**: Patient-Derived Xenografts (PDXs) model; **C**: Patient-Derived Tumour Organoids (PDTOs) model

Cancer organoids are revered for their ability to retain the heterogeneity and fundamental morphology of patient’s tumour [4]. This was not realized by two-dimensional (2D) cell culture lines, the current model used for in vitro cancer modelling and drug screening [5]. 2D cell cultures have been vital in cancer research, but, their main limitation lies in their inaccuracy in replicating cancer cells in vivo [6]. Their 2D structures causes changes in polarity, morphology and method of division as well as disturbances in interactions between the cellular and extracellular environments. Most importantly, they are unable to accurately recapitulate the complex and dynamic nature of cancer, especially drug resistance mechanisms which remains the principal limiting factor to achieving cures in patients with cancer [7]. Fundamentally, they are inaccurate representations of in vivo tumours, but are used widely due to their ease of proliferation, low-cost maintenance, amenability to performance of functional tests [8] **(**Fig. 1A**).**

Another promising cancer model is the patient-derived xenografts (PDXs). PDXs are able to diligently recapitulate the biological characteristics of the human tumour, but are extremely time consuming and expensive to utilize [9]. Furthermore, PDXs also demonstrate the ability to undergo murine-specific tumour evolution, [10] and raises various ethical concerns regarding the use of animal models for experimentation [11]. For these reasons, PDXs are unsuitable for high-throughput drug screening (HTS) and remain largely in the laboratory for research. (Fig. 1B).

As a result, tumour organoids, for their ability to reconcile the shortcomings of current cancer models holds great promise for optimization of preclinical drug discovery. Tumour organoids are less expensive, time-consuming and resource-intensive than PDXs [12]. Furthermore, tumour organoids are a suitable model which both, reflects the physiological features of an actual patient’s cancer [13] as well as are compatible with the standard procedures in HTS drug screening in the pharmaceutical industry **(**Fig. 1C).

While the utility of tumour organoids in preclinical drug discovery and screening have been established, there have only been marginal progress made in clinical application. Despite their posited benefits and enormous potential preclinically, the clinical translation for the organoid models in cancer therapy remains to be elucidated. Until this link is established, cancer patients are unlikely able to benefit from tumour organoid technology for the purposes of functional precision cancer medicine.

Most reviews in the literature have focused on the use of organoids as an alternative model for human cancer in the context of preclinical drug screening and development [14] or highlighted its benefits as opposed to 2D cell lines and PDXs [9]. However, clinical applications of PDTOs have been largely neglected, resulting in a gap in the current research. Researchers are engrossed on the discovery and refinement of techniques in growing different types of organoids and its applications in preclinical research, but fail to realize the various obstacles for use in real-life patients. While the general premise is that organoid technology can be potentially used for all patients, this is not true, despite the current advancements in the field. As organoids have been around for more than a decade, it is finally time to look towards its liberation from the laboratory to benefitting cancer patients at the bedside.

In this review, we seek to explore the main reasons for bottlenecks in the clinical translation of PDTOs, an important top-down tumour organoid model which is directly established from clinical cancer biopsies in a tissue-specific fashion. Additionally, we discern the groups of patients to recommend this technology for as well as propose solutions to bridge the gap from bench to bedside.

## Patient-derived tumour organoids (PDTOs)

A widely accepted definition of “organoids” is an in vitro 3D structure, developed from stem cells and consisting of organ-specific cell types that self-organize via cell sorting and spatially restricted lineage commitment in a manner similar to in vivo, to recapitulate tissue or organ functionality [13, 15]. Organoids are derived from two main sources, adult stem cells (ACS) or pluripotent stem cells (PSCs) through processes akin to human organogenesis. [15, 16] Organoids have been successfully established in normal human colon [17, 18], liver [19], pancreas [20], prostate [21, 22], stomach [23], fallopian tubes [24], taste buds [25], salivary glands [26], oesophagus [27], lung [28], endometrium [29] and breast. [30] The culture protocol used to establish these organoids were based on the work of Sato et al. [31], demonstrating that 3D epithelial organoids can be grown from a single leucine-rich repeat-containing G protein-coupled receptor 5 (LGR5) + intestinal stem cell.

Engineered tumour organoids are normal organoids which are gene-edited to be mutated into tumour organoids [9]. A combination of CRISPR-Cas9 gene editing and organoid culture is used to derive tumour organoids. Manato, M et al. demonstrated that targeting induction of driver mutations in APC, SMAD4, TP53, KRAS and/or PIK3CA in healthy intestinal organoids could model the genesis of an adenoma. But these driver mutations alone were not sufficient to induce tumorigenesis [32].

Patient-derived tumour organoids (PDTOs) are tissue-derived tumours from patients via surgically resected specimens,biopsied tissues or circulating tumour cells that are able to grow into tumour organoids after embedment into a 3D matrix [9]. The culture protocol formulated by Sato et al. [31] would also form the basis for cultivation of PDTOs. There have been 12 types tumour organoids established with good results. (Table 1) Phenotypic and genotypic profiling of PDTOs revealed that they were similar to the original tumours and retained the same gene-mutation spectrum. Studies have also shown that PDTOs can recapitulate the biological characteristics of primary tumours including histological complexity and genetic heterogeneity of cancer [33]. Engineered tumour organoids are used generally to understand the detailed process of genetic mutation in carcinogenesis and not for drug screening or clinical applications. Hence, for the purposes of this review, only PDTOs will be discussed for its clinical applications.

**Table 1:** Table showing list of established Patient-Derived Tumour Organoids (PDTOs)

System	Cancer Type	Success Rate of PDTOs	Reference
**Digestive**	Pancreatic Cancer	62% (52/83)	[34]
		75% (103/138)	[35]
		85% (17/20)	[36]
			[20]
	Colorectal Cancer	100%	[37]
		~ 90% (22/27)	[38]
	Hepatocellular Carcinoma	26% (10/38)	[39]
		100% (13/17)	[40]
	Gastric Carcinoma	50%	[41]
		71% (10/14)	[42]
	Metastatic Gastrointestinal Carcinoma	70% (> 100)	[43]
		76% (13/17)	[44]
	Esophageal Carcinoma	31% (10/32)	[45]
	Appendiceal Carcinoma	75% (9/12)	[46]
**Respiratory**	Lung Carcinoma	88% (n = 16)	[47]
	Non-Small Cell Lung Cancer	71.43% (10/14)	[48]
	(Primary & Metastatic)	100% (3/3)	[49]
		28% (n = 18)	[47]
	Mesothelioma	100% (2/2)	[50]
**Urinary**	Prostate Cancer (Primary & Metastatic)	16% (4/25)	[51]
		18% (6/32)	[52]
	Bladder Carcinoma	70% (12/17)	[53]
	Renal Cell Carcinoma	74% (25/35)	[54]
**Reproductive**	Breast carcinoma	~ 80% (> 155)	[30]
	Endometrial Carcinoma	100% (15/15)	[29]
	Ovarian Cancer	65% (n = 32)	[55]
**Nervous**	Glioblastoma	91.4% overall	[56]
		66.7% (IDH1 mutant)	
		75% (recurrent)	

## Potentials and challenges of organoids in precision cancer medicine

While PDTOs have great potential for profound advancements in cancer medicine, it is important to recognise the fundamental challenges that exist when adopting this technology. In this section, we discuss the various advantages and disadvantages of using PDTOs as well as take a look at the present attempts at utilizing PDTOs in clinical trials.

### Advantages and disadvantages of organoids in drug screening

Interpatient heterogeneity with respect to sensitivity to anti-cancer drugs in clinical use have been largely disregarded by most studies using 2D established cell lines. Drug development is performed under the premise that those cancers of the same histopathology respond in the same way to a drug [13]. This is assumption is one that has far-reaching impacts on the current standards of treatment, with only certain patients responding to treatment.

Therefore, precision cancer medicine is needed. It essentially means identifying the treatment (s) that would best decrease tumour size or eradicate the patient’s cancer with the least adverse side effects. It is notable that precision cancer medicine today is nearly interchangeable with genomic medicine. However, the reliance of precision medicine to define specific genetic abnormalities as targets for drugs is inherently limiting and highlights a major weakness of precision cancer medicine [51]. Therefore, functional precision cancer medicine, is needed to identify new drugs and assign existing drugs to larger numbers of patients with cancer. It is via functional precision cancer medicine that organoids will have clinical applications in real life.

While organoids have many benefits, their limitations have also been discussed extensively in literature. Intrinsically, organoids are still considered imperfect reproductions of in vivo cellular conditions. They are unable torecapture the complicated structures of the tumour microenvironment (TME) such as the surrounding mesenchyme, blood vessels, immune cells and neurons [14]. Tumour progression and drug resistance of cancers are influenced by the components of the TME, such as surrounding fibroblasts and immunocytes [57]. For example, in breast cancer, cancer-associated fibroblasts are present in high numbers in the TME and enhances metastasis of both premalignant and malignant mammary epithelial cells [58]. As a result, organoids only partially recapture the complex disease process of carcinogenesis.

Next, for aggregation of organoids into 3D structures, the use of Matrigel or another animal-based matrix extract is required. However, these are composed of complex components consisting of undefined growth factors that could potentially affect cellular activities [57]. This causes undesirable variability and may affect reproducibility of organoids [59]. Furthermore, with a relatively rigid extracellular matrix, they could limit drug penetration, which hampers its use in drug screening [60].

Organoid generation is more time-consuming and resource-intensive than traditional 2D cell lines. They are technically difficult to generate and require trained personnel to prepare the primary cells from patient’s tissue [13]. Furthermore, organoid production relies on embedment of stem cells into Matrigel restricts the surface to mass ratio, thereby limiting the production of organoids in a large scale [4]. While still compatible for high throughput drug screening (HTS), it is inferior than 2D cell lines in this aspect.

Another limitation of organoids is that they lack vascularization, which limits the maximum size of organoids produced. The determinant of organoid size is the maximum distance that oxygen and nutrients can diffuse inside the organoid as they lack vascularization [4, 61]. As the organoid increases in size, there is a resultant oxygen gradient leading to limited availability of oxygen and eventual death of the cells in the center of organoids [62].

Tumour size at diagnosis is frequently used to estimate prognosis. Larger tumours are often correlated with increased metastatic risk. This can be attributed to the fact that that given a certain mutation rate, size becomes a key factor in predicting the presence of drug-resistance mutations [7]. Mathematical models of cerebral organoids show that 1.43 mm is the maximal attainable size, [61] which may be significantly smaller than the original tumour size. As a result, drug-resistance mutations may not be present in in-vitro tumour organoids which differs from the actual parental tumour.

Finally, organoids also raise many ethical concerns which need to be dealt with carefully. This includes the use of human embryos and the development of biobanks which can be stored and expanded indefinitely, which raises concerns regarding informed consent and ownership [63].

Despite its limitations, organoids are still regarded as an upgrade from traditional 2D cell lines. Their ability to mimic tumour morphology and heterogeneity is one that holds great potential for a wide array of applications. However, the extent to which organoids can recapitulate the heterogeneity of tumours are still largely undetermined [64]. Extensive passaging of organoids can result in loss of heterogeneity due to cellular adaptation to culture conditions in vitro by epigenetic or genetic mechanisms [65]. With its principal trait still under contention, organoid technology is still a long way from having real-life clinical impact.

### Clinical trials

A comprehensive search for past and ongoing clinical trials pertaining to PDTOs was conducted on ClinicalTrials.gov (https://clinicaltrials.gov/). The search was conducted in January 2022 and included the search terms and results as shown in (Table 2).

**Table 2:** Primary search strategy for clinical trials involving PDTOs and cancer

Term	Synonym	Term	Related Words	Search Results	Relevant	Duplicates
Cancer	Neoplasm Tumour Oncology Neoplastic Syndrome Malignancy Neoplasia Neoplastic Disease	Organoids	Tumour Organoids	90	74	-
			Patient-derived OrganoidsPatient-derived tumour organoids			
		3D cell line	Cultured cells	42	2	1
			Cell lining			
			Three dimensional			
			3 dimensional			
		3D cell culture	Culture cell	7	4	3
			Cellular			
			Three dimensional			
			3 dimensional			
		3D cell model	Modeling system	51	11	11
			Cellular			
			Three dimensional			
			3 dimensional			

The search generated an initial list of 190 registered clinical trials, which was subsequently reduced to 76 after a screen for relevance to PDTOs and removal of duplicates. The number of clinical trials differentiated by type of cancer as well as stage of disease is summarized in (Table 3). Currently, the most common cancer types investigated in clinical trials are breast cancer, pancreatic cancer and colorectal cancer.

**Table 3:** Summary of number of clinical trials divided into cancer type and stage of disease

Type of Cancer	Early/ Locally Advanced (*n* =)	Refractory/ Metastatic (*n* =)	All Stages (*n* =)	Total Number of Studies	Percentage of Total Studies (%)
Breast Cancer	5	6	1	12	15.8
Pancreatic Cancer	3	3	4	10	13.1
Colorectal Cancer	3	3	3	9	11.8
Lung Cancer	2	2	4	8	10.5
Different Gastrointestinal Cancers	3	2	0	5	6.6
Esophageal Cancer	1	2	0	3	3.9
Biliary Tract Cancer	2	1	0	3	3.9
Kidney Cancer	2	1	0	3	3.9
Ovarian Cancer	0	2	1	3	3.9
Different Reproductive Cancers	1	2	0	3	3.9
Any Cancer Type	0	3	1	4	5.3
Liver Cancer	1	1	0	2	2.6
Glioblastoma	1	1	0	2	2.6
Neuroendocrine Carcinoma	0	1	1	2	2.6
Sarcoma	1	1	0	2	2.6
Different Head and Neck Cancers	1	0	1	2	2.6
Multiple Myeloma	0	0	1	1	1.3
Prostatic Cancer	0	1	0	1	1.3
Bladder Cancer	1	0	0	1	1.3

However, most clinical trials (68 out of 76) discussed PDTOs in a preclinical context for purposes such as assessment for identicality with parental tumour for histopathological and genetic information, long term expansion for biobanking, discovering baseline establishment rate of PDTOS, drug testing with subsequent clinical correlation, development of culture medium and discovery of novel biomarkers.

Only 8 clinical trials investigated the use of PDTOs in the context of functional precision medicine via drug sensitivity screening (Table 4). This small number could likely suggest the great potential for PDTOs in a functional drug screen, but with several limitations in practical application. No results have been published from these trials, with 5 in the recruitment phase, 2 not yet recruiting and the status of 1 being unknown. Breast cancer, pancreatic lung cancer, bladder cancer and squamous cell carcinomas of the head and neck, colorectal cancer and ovarian cancer are the cancer types investigated in these 8 trials. It is notable that 3 out of 8 trials were on breast cancer, likely due to its high incidence in women as well as limited options in treating aggressive subtypes such as triple-negative breast cancer.

**Table 4:** List of clinical trials investigating PDTOs for functional precision testing

**Gastrointestinal System**									
**Pancreatic Cancer**									
	**Identifiers**	**Status**	**Stage of Cancer**	**Histology**	**Method of Tissue Acquisition**	**Drugs**	**Life Expectancy**	**Inclusion Criteria**	**Exclusion Criteria**
1	NCT04931394	Recruiting	Early	- Pancreatic Carcinoma - Pancreatic Adenocarcinoma - Mucinous Adenocarcinoma - Adenosquamous Carcinoma	Surgical Resection	Gemcitabine, 5-fluorouracil, Paclitaxel, Oxaliplatin, Irinotecan	> 90 days	Complete R0 resection for pancreatic cancer with no evidence of malignant ascites, peritoneal metastases or distant metastases	Cannot tolerate targeted chemotherapy and targeted therapy
									Severely Impaired Organ Function
2	NCT04931381	Recruiting	Locally advanced/ metastatic	- Pancreatic Carcinoma - Pancreatic Adenocarcinoma - Mucinous Adenocarcinoma - Adenosquamous Carcinoma	Core needle biopsy	Gemcitabine, 5-fluorouracil, Paclitaxel, Oxaliplatin, Irinotecan	> 90 days	Patient must have a tumour lesion that is amenable to a core needle biopsy	Cannot tolerate targeted chemotherapy and targeted therapy
									Severely Impaired Organ Function
**Breast Cancer**									
3	NCT04450706	Recruiting	Metastatic	HER2-negative Breast Cancer	Tumour Biopsy	Docetaxel, Cyclophosphamide, Adriamycin, Methotrexate, 5-fluorouracil, Paclitaxel	> 6 months	Metastatic or recurrent unresectable breast cancer:	Unable to undergo biopsy safely
								Triple-negative breast cancer without prior treatment in the metastatic setting	Severely Impaired Organ Function
								Willing and able to undergo a baseline biopsy. Safely undergo tumour biopsy	Diagnosis of any other malignancy within 2 years
								Successful acquisition of a tissue sample containing ≥ 20% tumor content	
4	NCT03544047	Unknown	2–3	Breast Cancer	Surgical Resection, Tumour Biopsy	Paclitaxel, Trastuzumab	> 6 months	No prior treatment	Unable to obtain sufficient tumor organizer by operation or biopsy
								According to the RECIST standard, the lesion was measured (the diameter of the primary lesion was greater than 1.0 cm or the short diameter of the lymph node was greater than 1.5 cm)	History of other malignancies
								Metastatic lesions or primary lesions can obtain surgical tissue or adequate biopsy tissue	Severely Impaired Organ Function
5	NCT05177432	Not yet recruiting	All	Breast Cancer of any subtype	Tumour Biopsy	10–12 anti-cancer drugs (Alpelisib, transtuzumab-emtansine and others not specified)	> 12 weeks	• At least 1 tumour lesion (primary or metastatic) amenable to fresh biopsy • At least 1 measurable tumour lesions based on RECIST 1.1 criteria • Has documented progressive disease from last line of therapy • Has received at least 1 line of palliative systemic therapy	• Male Breast Cancer • Pregnancy • Secondary Primary Malignancy • Contraindication to anti-cancer therapy in drug screening panel • Treatment within last 30 days with any other drug Concurrent administration of other tumour therapies
	**Respiratory System**								
	**Lung Cancer**								
6	NCT05136014	Enrolling by Invitation	All	Lung Cancer Lung Adenocarcinoma EGFR Activating Mutation KRAS Mutation-Related Tumors Non Small Cell Lung Cancer	Surgical Resection	Osimertinib	> 30 days	With non small cell lung cancer of any stage undergoing surgical resection at the Nancy University Hospital	• Hepatitis • HIV •Pregnancy
	**Urinary System**								
	**Bladder Cancer**								
7	NCT05024734	Not yet recruiting	Early (non muscle invasive)	intermediate risk non muscle-invasive urothelial carcinoma of the bladder (pTa low grade)	Tumour Biopsy	Epirubicin Mitomycin Gemcitabine Docetaxel	> 24 months	• Histologically confirmed intermediate risk non muscle-invasive urothelial carcinoma of the bladder (pTa low grade) Patients Representative fresh tumor specimen for PDO generation and drug screen	• Known previous high grade and/or high risk non muscle-invasive bladder cancer • Previous Intravesical biological/immuno (BCG) therapy • Severe infection within 4 weeks prior to cycle 1, day 1 • Contraindication for frequent catheterization Voiding dysfunction
	**Different Cancers**								
	**Head and Neck, Colorectal, Breast, Ovarian Cancer**								
8	NCT04279509	Recruiting	All	Histological or cytological diagnosis of head and neck squamous cell carcinoma (HNSCC), colorectal, breast or epithelial ovarian cancer	Tumour Core Biopsy, Blood Sampling	5-fluorouracil, carboplatin, cyclophosphamide, docetaxel, doxorubicin, gemcitabine, irinotecan, oxaliplatin, paclitaxel and vinorelbine. etoposide, ifosfamide, methotrexate, pemetrexed and topotecan	> 12 weeks	At least 1 tumour lesion (primary or metastatic) amenable to fresh biopsy	Pace of cancer progression requiring commencement of anti-cancer therapy within 4 to 6 weeks
								At least 1 measurable tumour lesions based on RECIST 1.1 criteria	Severely Impaired Organ Function
								Able to wait at least 4 to 6 weeks before initiating the next line of anti-cancer therapy	
								Has received at least 2 lines of palliative systemic therapy	

Methods of tissue acquisition included surgical resection, core needle biopsy as well as blood sampling for circulating tumour cells (CTCs), all largely dependent on stage of disease. Fluorouracil, Docetaxel and Paclitaxel were the most frequently screened anti-cancer drugs, likely attributed to the fact that these were regularly used to treat a range of different cancers. The minimum life expectancy for patients was at least 90 days, which may be considered insufficient when integrating the turnaround time for a clinical organoid drug screen. Furthermore, inclusion criteria such as being able to delay initiation of therapy for a minimum of 4 to 6 weeks may hinder treatment as a narrow therapeutic window exists for optimal results.

It is apparent that more efforts should be directed towards establishing proper guidelines to govern clinical trials on precision therapy. Titration of the appropriate parameters such as life expectancy, total time taken for a clinical organoid screen and patient factors should be considered.

## Bottlenecks of patient-derived tumour organoids for clinical application

With the potential to be at the forefront of precision medicine, PDTOs could signify the dawn of customizable therapies for cancer patients. Despite being present for more than a decade, PDTOs have yet to debut as a clinically relevant model.

References were searched and retrieved from the database PubMed. A twofold search strategy which consisted of: a primary search to identify studies related to cancer using “organoids (MeSH Terms) and cancer (MeSH Terms); a secondary search using “organoids (MeSH Terms) and neoplasms (MeSH Terms)” was conducted. Supplementary searches were performed when necessary to retrieve additional information.

**Fig. 2:**
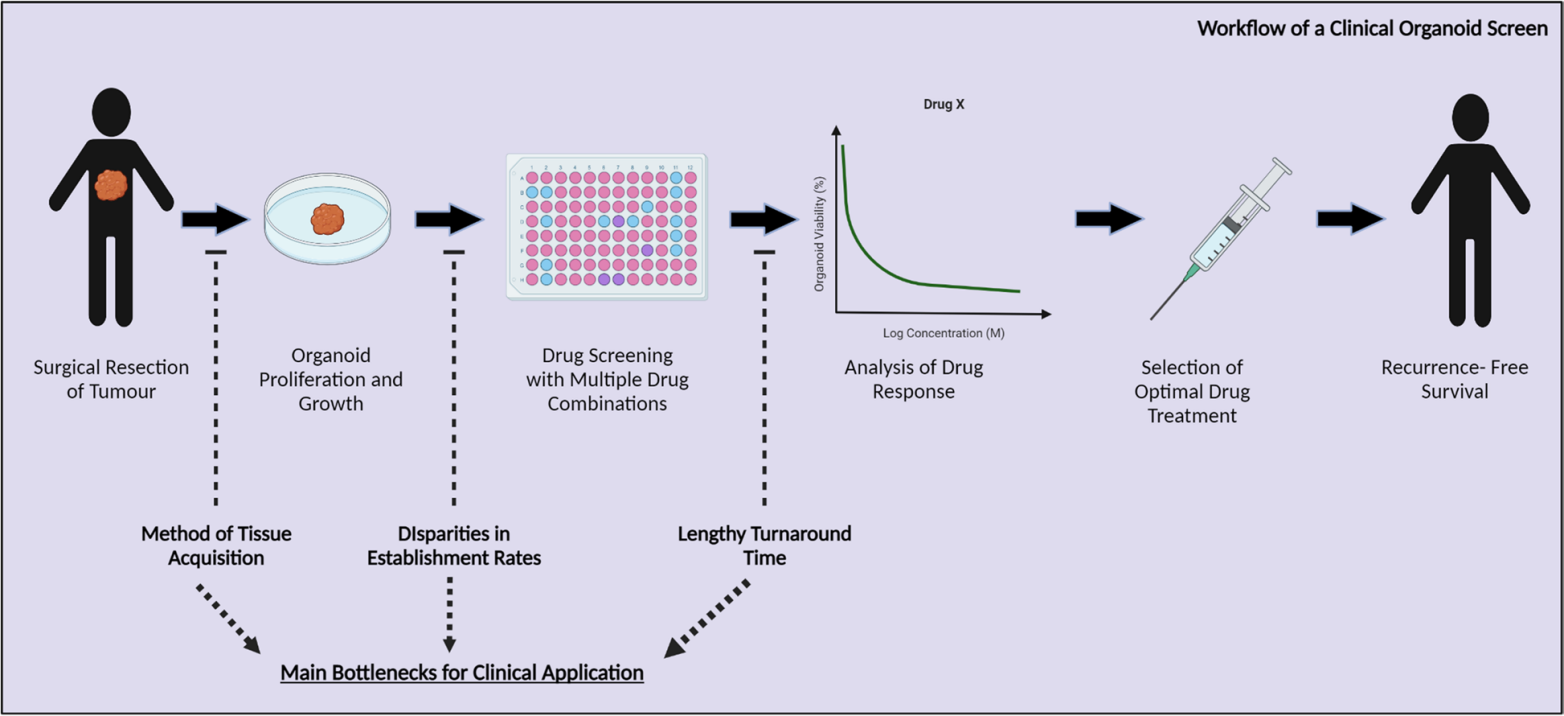
Main bottlenecks in the workflow of a clinical organoid screen

In the following section, we will discuss the main bottlenecks identified which prevent the transition of PDTOs from bench to bedside (Fig. 2).

### Tissue acquisition

PDTOs are established from surgically resected tissue, biopsied tissues or circulating tumour cells [9]. While PDTOs have been successfully established from primary and metastatic tumours, there are several key factors to consider.

Firstly, attempts to create tumour organoids from biopsies have low overall success rates for indefinite propagation and expansion due to inability of cells to adapt to in vitro conditions quickly enough to avoid senescence. Needle biopsies of metastatic lesions especially bone, have scant starting material, limiting the cell to cell interactions for viability of tumour organoids to survive [51]. This would likely translate into the comparatively lower establishment rates of tumour organoids from needle biopsies of metastatic sites as opposed to primary tumour. Therefore, the optimal way to acquire tissue for tumour organoid growth is via a surgical resection as this provides sufficient starting material for good propagation of the tumour organoids.

Secondly, we have to consider the sites in which to obtain tissue for PDTO establishment, especially in the context of metastatic cancer. It is insufficient to obtain tissues from the primary site alone as there are genomic differences between primary and metastatic tissues. Metastatic cells acquire new genomic features which allow for separation from original tissue and avoidance of anoikis, to enter the lymph or blood vasculature to localize to the new tissue for colonization to form a new tumour [66]. As such, metastatic tissue significantly expresses more alterations of specific genes which induce high metastatic ability compared to the primary tumour. Evidence has shown varying degrees of concordance between the genetic makeup of metastatic sites and their primary tumours amongst multiple types of solid cancers [67, 68]. Furthermore, comparisons of different metastases often reveal substantial levels of heterogeneity [69, 70]. As a result, tissue should be acquired from the primary tumour as well as all metastatic sites to reflect the composite mutational landscape for drug screening [71].

However, patients with metastatic disease are likely not amenable for curative treatment via surgical resection and thus raises many questions as to their suitability for tumour organoid technology. Common sites for metastasis often involve nearby lymph nodes and organs including the liver, lung, bone and brain [72]. Therefore, in patients with metastatic disease, they are to be subjected to surgical resections in multiple sites for the establishment of tumour organoids and not for curative purposes. While there have been successful attempts of establishing tumour organoids via needle biopsies, [51] the ideal starting material would be attained via surgical resection.

Lastly, with extensive passaging of organoids resulting in loss of heterogeneity due to cellular adaptation to culture conditions in vitro, [65] it raises concerns as to whether tumours organoids are able to accurately recapture ongoing disease process in patients for the purposes of real time monitoring of drug response. Whether repeat biopsies of tissue should be taken and whether tissue would be sufficiently available after initiation of treatment would need to be considered as well..

### Disparity in establishment rates

PDTOs have been successfully established from a range of different cancers. (Table 1) However, there exists a huge disparity in success rates, ranging from 16 to 100%. [49, 51].

The large difference in success rates can be attributed to various reasons. Firstly, a crucial factor for failure of establishment is via contamination by epithelial organoids [14]. Tumour organoids grow at a slower rate than normal epithelial cells and are often out competed by them [73]. This is a problem which plagues all organoid cultures despite effortsto refine the tissue extraction process to minimize contaminating cells.

Secondly, there are no defined culture conditions to grow specific tumour organoids. At present, the culture protocol used to grow tumour organoids are based largely on the work of Sato which was initially used to grow benign intestinal organoids form Lgr5 + intestinal stem cells [31]. There have been no validated culture mediums for establishment of tumour organoids and researchers made modifications to culture protocol for their own use by including compounds were hypothesized to support growth or factors that were shown to support other types of tumour organoids [55]. These adjustments to the culture media are based on “informed guesses” by the researchers which may have negative impacts on tumour organoid growth. Notably, Van de Wetering found that rare subtypes of colonic tumours were not amenable to published culture protocol [38]. To add on, studies have also shown that medium composition exhibits selective pressure on PDTOs and may influence the genetic composition of cancer organoids via selection against certain tumour subclones [37]. These factors may lead to a failure of establishment of tumour organoids or an establishment of tumour organoids which are not representative of the original tumour, resulting in non-physiological responses when subjected to drug screening.

Thirdly, there is a lack of standardized protocols for the establishment of PDTOs. This can lead to batch-to-batch variation and an overall lack of quality control between and within research institutions [74]. Furthermore, PDTOs are technically challenging to establish, requiring trained personnel to process and prepare the cells from the patient’s tissue [13]. All these can lead to differing rates of success in establishing PDTOs and can result in a limited reproducibility of tumour organoids from the same subtype. Drug screening results would also be difficult to interpret due to institutional variations.

With huge differences in establishment rates, it will not be feasible to recommend this technology for cancer patients who require reliable answers in a timely manner. Until there is an increase in success rates of establishment, it would be a challenge for tumour organoids to be used inclinical practice.

### Lengthy timeline

To prevent tumour progression and upstaging, treatment should be initiated promptly upon diagnosis of cancer. Delays in cancer treatment can lead to poorer outcomes and require more aggressive treatments with unnecessary morbidity and mortality [75]. It has been found that just a 4-week delay in treatment is associated with an increase in mortality across all common forms of cancers- bladder, breast, colon, rectum, lung, cervix, and head and neck [76]. PDTOs can take 4–6 weeks for successful establishment, that is, reaching a minimum volume of 400um across before initiation of drug screening [77]. Clinicians would have to consider how organoids can be integrated into the time sensitive nature of cancer and make sure patients would not miss the best therapeutic window if it were to be used for the purposes of drug screening.

Negating the differing rates of successful establishment of tumour organoids, there still exists a turnaround time for an organoid screen using anti-cancer drugs. The reported time for a drug screen on gastric cancer organoids is less than 2 weeks [41], but this is largely dependent on the number of drugs to be tested. Different combination chemotherapy regimens, approaches to dosing intensities and shorter-interval administrations of chemotherapy have shown to improve the success rates of treatment by preventing early regrowth of tumours [7]. The permutations of combination drugs as well as interval dependent strategies to model real-life chemotherapy cycles may potentially extend the turnaround time for an organoid drug screening.

Next, there is no established repertoire of drugs to be screened for each type of cancer, contributing to the ambiguity of the total expected time frame for a cancer organoid drug screen. In cancers with limited effective 1^st^ line drugs for therapy like hepatocellular carcinoma, there is a need to screen beyond the conventional range of drugs available. Many patients with hepatocellular carcinoma (HCC) often present with advanced disease, which is unsuitable for surgical resection- one of the mainstays of therapy [78]. Therefore, systemic therapy is would be considered the most appropriate option. For HCC, only sorafenib and regorafenib, oral multikinase inhibitors that that block RAF signalling have been proven to improve survival rates marginally by 3 months [79, 80]. However, the 5-year survival rates for patients with Stage II and III disease are still low, at 37 and 16% respectively [81]. Therefore, efforts should be made to discern the range of drugs to be screened for each type of cancer, specifically in circumstances where there are limited effective therapies. With more than 100 available chemotherapy drugs and the prospects of drug repositioning to find new applications for existing drugs, there exists a wide variety of drugs to screen [82].

## Potential strategies

**Fig. 3:**
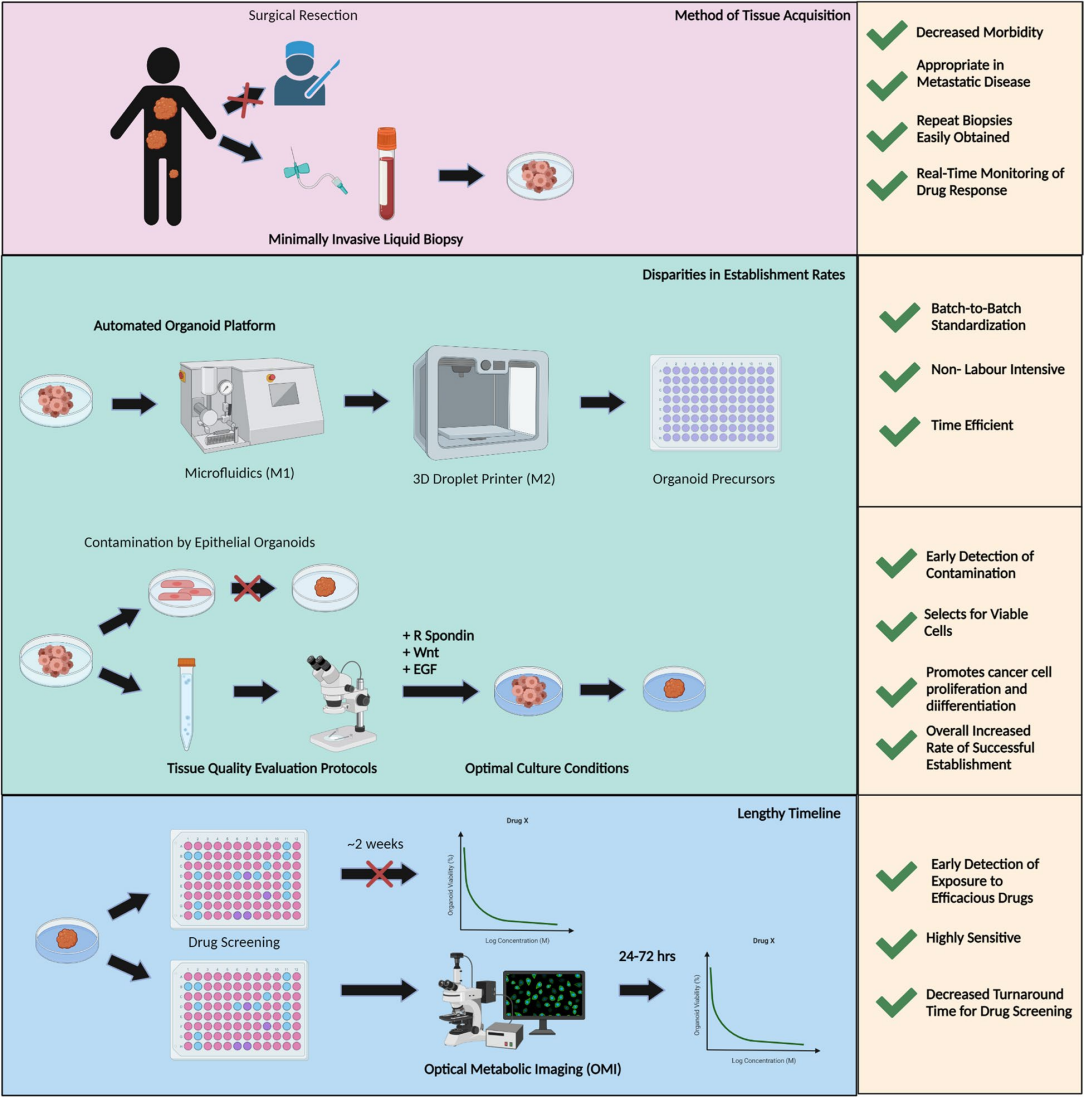
Potential solutions to overcome the bottlenecks in transitioning from bench to bedside

In this section, we discuss the potential solutions to overcome the main bottlenecks in transitioning PDTOs from bench to bedside (Fig. 3).

### Circulating tumour cells as viable surrogate resource for development of PDTOs

Current methods to obtain starting material for tumour organoids necessitate a minimum of 1cm^3^ of tissue, which are routinely extracted via surgical resection specimens or core needle biopsies. However, the latter yields low establishment rates and hence, an ideal method for tumour organoid isolation from patients would be via surgically accessible tumours. This limit the use of this technology for patients with disseminated metastatic disease as it is not amenable to surgical resection. Liquid biopsies may represent a potential solution to overcome the physical restrictions of tissue biopsies.

Minimally-invasive liquid biopsies as a surrogate to obtain tumour material has several advantages. Firstly, the morbidity associated with tissue biopsies such as the inherent risks and surgical complications can be avoided. Risks such as tumour seeding to other sites can eradicated [83]. Secondly, tumours located in technically difficult areas such as head and neck carcinomas can be sampled. Lastly, in patients with multiple sites of malignancy, the practical implications of multiple biopsies can be negated.

Circulating tumour cells (CTCs) are rare subsets of cells found in the blood of patients with solid tumours, shed from primary tumours or metastatic lesions into the vasculature to initiate metastatic lesions at distant sites [84]. CTCs recapture primary tumour heterogeneity, mimic parental tumour properties and most importantly, are obtained simply via a peripheral blood sample of 7.5 ml [85]. CTCs represent an alternative to replace traditional tissue biopsies in acquisition of starting material for tumour organoids which may be technically challenging and impractical in certain groups of patients [86]. They provide valuable information on the genetic landscape of malignancies in the body as well as track evolutionary dynamics of tumours [87]. Due to their minimally-invasive nature, repeat biopsies can be easily done for the purposes of serial monitoring, which facilitates the real time monitoring of disease progression and treatment outcomes in patients [88]. This is especially important in early detection of therapy resistance shortly after initiation of treatment, when a re-biopsy would be impractical due to the morbidity or lack of sufficient tissue available for initiation of tumour organoid growth.

CTCs have been successfully developed into prostatic cancer organoids, where evidence has shown that they closely resemble the histological traits of the primary carcinoma. The genetic mutations in the parental tumour such as PTEN loss, TMPRSS2-ERG interstitial deletion, SPOP and FOXA1 mutations, and CHD1 loss, were replicated as well [52]. Similar studies developing breast cancer organoids from CTCs have demonstrated that parental tumour characteristics were maintained [89].

While liquid biopsies represent a great alternative to tissue biopsies, it is important to note that CTCs are inherently rare, and challenging to isolate from patient blood samples [84]. As new techniques such as microfluidic chip systems and nanotechnologies emerge to enrich and isolate CTCs, we can look towards the use of CTCs for development of PDTOs in the near future.

### Automation and techniques to improve establishment rates

Disparities in establishment rates of PDTOs can be largely attributed to the techniques used in laboratories to grow organoid culture. The lack of standardization due to the high reliance on manual labour required for organoid growth leads to significant batch-to-batch and organoid-to-organoid variability [90].

An automated organoid platform, to rapidly generate uniformed organoid pre-cursors, poses a superior alternative to the labour-intensive and time-consuming method traditionally used to generation organoids. The model consists of two modules in synchronization, a microfluidics-based module (M1) for organoid production and a 3D droplet printing module (M2) for automated organoid distribution [91]. Besides being able to regulate the quality of organoids produced, automation is able to reduce the entire duration of a clinical organoid screen to just 1 week, from tumour sampling to recommendation of treatment. Organoids derived via automation have been shown to recapitulate parental tumour heterogeneity, genetic and mutational profiles and most importantly, interpatient heterogeneous responses to anticancer drugs.

Other methods to improve establishment rates include techniques to minimize contamination in PDTO culture. Before initiation of culture, sequential refinement of tissue extraction processes and tissue-quality evaluation protocols should employed. Dissociation of cancer tissues to form an aliquot of cell suspension for histological examination to select for viable cells can minimize contaminants, especially epithelial organoids [92, 93].

Another avenue to promote successful organoid growth would be the development of an appropriate culture system to sustain the various needs of the different stages of organoids. Culture protocols should be defined for all subtypes of cancer organoids and should include growth factors that stimulate cell proliferation and differentiation as well as inhibiting apoptosis. For example, R-spondin, Wnt and Epidermal growth factor (EGF), known as the “troika for organoid culture” have been found to promote and maintain organoid growth for extended periods of time [73, 94]. Recent studies have shown that Fibroblast growth factor 7, Noggin, Neuregulin1, Y-27632, A83-01 and SB202190 are suitable for breast cancer organoid growth [95]. As more efforts are made to design and optimize culture systems to support organoid culture, we can expect the establishment rates of PDTOs to improve to accelerate their clinical translational research and application in prospective clinical trials.

### Optical metabolic imaging of PDTOs

Differences in drug-screening protocols such as drug exposure timing, minimum organoid size required and timing of treatment relative to the seeding of organoids contribute to its ambiguity. Some researchers exposed organoids to drugs immediately after plating while others let the organoids recover for a few days before commencement of treatment [35, 96]. Average readout is done 1 week after initiation of the drug screening,, but can range from 1 to 24 days of exposure to drugs [97].

Optical Metabolic Imaging (OMI), a microscopy technique sensitive to changes in cellular metabolism allows for measurement of drug-induced change in cellular metabolism, can potentially reduce the total drug turnaround time when used as an adjunct to drug response monitoring [98, 99]. OMI is able to detect metabolic changes within the first 24-72 h of exposure to efficacious drugs, decreasing various parameters such as drug exposure time and time lapse to readout [100]. Research has shown that OMI is highly sensitive and can enable early reporting of drug treatment efficacy in tumour organoids [101]. When used in breast cancer organoids, OMI measurements of drug responses corresponded with that of HER2 or Estrogen Receptor status in the original tumour [99]. When used in the context of initiation of time-sensitive cancer therapies, these are encouraging results showing that OMI is an excellent tool to accelerate the workflow of a clinical organoid drug screening.

## Concluding Remarks and Future Perspectives

As PDTOs transition gradually to the bedside, other concerns will start to arise. The potential cost, ethical concerns and associated risks of PDTOs should be defined. The cost of growing PDTOs of different cancers are not standardized, the culture protocol varies for different cancers due to different materials required as well as total time taken for successful establishment. The cost of screening a range of different drugs should be factored in as well. While still undefined, the consensus isthat growing 3D cell cultures are more expensive and resource-intensive than 2D cell culture [8]. When translated to clinical practice, patients with a lower socio-economic status may have limited accessibility to such technology. It would be a challenge to balance the ethical and fiduciary responsibilities in the pricing of PDTOs as they are likely to be grown in private laboratories. Furthermore, PDTO drug screening comes with its associated risks and potential for failure. Patients have to be aware of the disparities in establishment rates between PDTOs, the potential risk for morbidity and mortality associated with tissue acquisition as well as potential treatment failure despite prior drug screening via PDTOs. All these are factors in which patients have to consider before being subjected to PDTO technology and would be the physician’s responsibility to access the suitability of such patients.

Furthermore, whether PDTOs are suitable for predicting treatment responses for radiotherapy is still not well established. In early-stage tumours, head and neck cancers are treated with radiation therapy as its sole therapy. Radiation therapy is also often used in combination with surgery and concurrent chemotherapy in advanced stages [102]. While there has been extensive research showing proof of concept in using PDTOs for chemotherapy drug screening, use of PDTOs to for radiotherapy response is still very underdeveloped. In particular, Pasch et. Al and colleagues demonstrated the use of PDTOs to predict sensitivity to a combination of chemotherapy and radiation, but more studies have to be done to determine its validity [103].

At this juncture, patients that are most suitable to benefit from PDTO drug screening are those without metastatic disease and mainstay treatment include surgical resection or chemotherapy. Patients would have cancers with high success rates in establishing PDTOs and would be able to accept the cost and associated risks of such technology. As technology advances, the potential for PDTOs to be universally adapted for use in all oncology patients in selection of the most efficacious treatment with the least side effects could be a reality.

The paradigm that functional medicine is unsophisticated or unrefined is one that has to be eradicated. The main criticism of functional medicine is that it identifies therapeutic opportunities without illuminating the underlying mechanisms. However, functional precision medicine fills a gap in translating PDTOs to the bedside in a more accelerated pace than purely genomic approaches to precision medicine.. Furthermore, functional precision medicine provides valuable information which is extremely relevant to understanding why effective drugs work even if their effectiveness is felt before we understand why it works. PDTOs are versatile, presenting us with a platform where researchers can understand the complex mechanisms of tumorigenesis, intra-tumoral heterogeneity and clonal evolution even when it is inefficient in translating that to clinical medicine.

As synergistic applications of organ-on-a-chip and 3D bioprinting are applied to organoids, the ability of organoids to be a comprehensive, encompassing cancer model which integrates the TME, microvascular network as well as various organs can be realized [104]. Organoids represent an exciting time in clinical research, where the resurgence of functional medicine can have immediate benefits to patients in the now. When its limitations are finally overcome, organoids could represent a new hope for cancer patients with limited options.

## Data Availability

Data sharing not applicable to this article as no datasets were generated or analysed during the current study.

## Abbreviations

3D: Three-Dimensional
AI: Artificial Intelligence
PDTOs: Patient Derived-Tumour Organoids
CTCsL: Circulating Tumour Cells
OMI: Optical Metabolic Imaging
WHO: World Health Organization
2D: Two-Dimensional
PDXs: Patient-Derived Xenografts
HTS: High-throughput Drug Screening
ACS: Adult Stem Cells
PSCs: Pluripotent Stem Cells
LGR5: Leucine-rich repeat-containing G protein-coupled receptor 5
TME: Tumour Microenvironment
CTCs: Circulating Tumour Cells
HCC: Hepatocellular Carcinoma
OMI: Optical Metabolic Imaging
